# Poor Mental Health Is Related to Excess Weight via Lifestyle: A Cross-Sectional Gender- and Age-Dependent Mediation Analysis

**DOI:** 10.3390/nu13020406

**Published:** 2021-01-28

**Authors:** Nathalie Michels

**Affiliations:** Department of Public Health and Primary Care, Faculty of Medicine and Health Sciences, Ghent University, 9000 Gent, Belgium; Nathalie.michels@ugent.be

**Keywords:** stress, depression, obesity, diet, physical activity, smoking

## Abstract

Within mental health as risk factor for excess weight, prevention-relevant questions remain: does the relation persist after considering lifestyle, which lifestyle parameters might be most important to target, which gender or age subgroups are most at risk? The cross-sectional Belgian health survey 2013 (*n* = 4687; ≥15 years) measured mental health via anxiety and depression symptoms (Symptom Check List 90-R) and distress (General Health Questionnaire-12). Logistic regression, multiple mediation and moderated mediation were applied. Poor mental health was significantly related to a higher excess weight prevalence (odds ratio (OR) = 1.18 with 95% confidence interval (0.17–1.19)) and an unhealthier lifestyle i.e., more smoking, sleep problems, disordered eating, soft-drink, and alcohol consumption; while less fruit/vegetables and physical activity and even lower snack intake. Associations were often gender- and age-specific e.g., poor mental health was only related to less snacking in men and middle-adulthood, while an association with more snacking appeared in youth (<25 years). Disordered eating, physical activity and smoking were significant mediators explaining 88% of mental-weight associations, after which the association became negative (OR = 0.92 (0.91–0.93)). Mediation by snacking and disordered eating was stronger in the youngest and mediation by smoking was stronger in women. Thus, especially youth has high mental health associated behavioral and weight risks and gender or age differences can explain conflicting literature results on lifestyle.

## 1. Introduction

In high-income countries, obesity, and mental disease rank highest towards disability-adjusted life years [1,2]. Indeed, psychosocial distress and the related negative affective state (anxiety or depression) are today widely present, e.g., psychological problems were reported by one third during the Belgian health interview survey in 2013 and these symptoms have been increasing since 2008 [3]. Interestingly, mental health and excess weight are bidirectionally linked to each other [4,5].

Insight in the behavioral mechanisms induced by poor mental health is essential for obesity prevention and intervention. Apart from a direct effect of the stress-induced cortisol hormone on abdominal fat [6], distress or mental health issues might indirectly change fat storage through lifestyle [4]. Herein, the main literature focus has been on stress-induced eating of unhealthy food items with excess calorie intake [7,8,9,10]. Apart from diet, stress or mental health issues may interrupt one’s choice to participate in healthy lifestyle behaviors such as physical activity [11] and adequate sleep duration or quality [12,13], while promoting unhealthy behaviors such as self-medication by alcohol overconsumption [14] or smoking [15,16]. Several of these lifestyle factors might mediate the relation towards weight change, e.g., diet-related factors such as emotional eating [17,18] and sleep [19] have been mentioned as mediators. Although multiple lifestyle factors play a role herein, only few studies [19,20,21] tested multiple mediation (i.e., independent mediation by several lifestyle factors at the same time) to identify the most important factor and whether a significant direct effect remained.

Still, literature on the effects of mental health on lifestyle is inconsistent. Indeed, psychological distress has been associated with both increased and decreased food intake [22], with increased and decreased physical activity [11,23], with increased and decreased alcohol consumption [14]. Hence, differential mental-adiposity associations may exist because of inter-individual differences in lifestyle and physiology. As part of inter-individual differences, ways of dealing with psychological distress have been reported to differ by gender and age in a large observational study [24], and biological gender differences in brain processes for emotions and reward behavior exist [25]. Concerning excess weight, most studies report stronger associations with mental health in women [4], but sometimes higher correlations were detected in men [26]. Thus, it is relevant to check age and gender moderation towards lifestyle and weight, to explain conflicting results and to detect high-risk populations for targeted prevention. Therefore, moderated mediation testing is needed but has seldom been implemented in this mental-lifestyle-weight research field [18,27]. Based on the few and sometimes conflicting studies examining gender/age differences, the hypothesis was that especially women are vulnerable to stress-induced eating and excess weight, while no strong a-priori hypotheses existed for the direction of moderation by age or for the other lifestyle factors.

This paper tests the relation of mental health with excess weight and lifestyle in a representative sample of the Belgian population. The question is whether the magnitude of disparity in overweight due to poor mental health would remain if unhealthy lifestyle risk factors are changed. The hypothesis is that poor mental health is related to excess weight, and that this is fully mediated by multiple unhealthy lifestyle parameters (i.e., more sleep problems, smoking, disordered eating, soft drink, alcohol, and snack consumption; while less fruit and vegetables intake and physical activity). Novel is the multiple mediation analysis testing all lifestyle factors together to detect overall and independent lifestyle mediation (e.g., is snacking still a mediator after considering other lifestyle parameters?) and the exploratory moderated mediation hypothesis (i.e., the possible mediation by lifestyle factors is gender- and age-specific), although the cross-sectional design does not allow causal statements.

## 2. Materials and Methods

### 2.1. Design

The Belgian Health Interview Survey is a Belgian nation-wide representative survey via multistage selection [28]. The data from 2013 was used since all relevant parameters were collected at that moment [29]. From 9114 participants ≥15 years, 4687 had full data for the current analyses since many had missing mental health data, i.e., when only a proxy reported about their health (they had no significantly different weight status, age, or gender). Based on a non-significant Little’s test (*p* = 0.268), the data was missing at random. The research protocol was approved by an ethical committee (University of Ghent, Gent, Belgium; 2012/0110). All procedures (including informed consent) were in accordance with the Helsinki declaration. The questionnaires were self-administered (exception: dietary intake and education was reported in-person) and available in Dutch, French, German, and English.

### 2.2. Mental Health

Depression (13 items; α = 0.89) and anxiety (10 items; α = 0.88) were measured by the Symptom CheckList SCL-90-R [30], validated for public health surveys [31]. Following questionnaire guidelines [30], the mean over all items of the subscale was taken (ranging from zero to four), and a score >1 was categorized as “at risk”. Anxiety and depression were taken together, as there was an overlap of more than 95%. Hence, the current article considered a person at risk if the anxiety and/or depression scale indicated at risk.

Psychological distress was assessed by the General Health Questionnaire (GHQ12) [32] detecting non-psychotic psychiatric problems in public mental health surveys with excellent discriminant validity [33]. The 12 items (e.g., “Felt constantly under strain?”; α = 0.83) had to be rated referring to the last 4 weeks: “not at all” (score 0), “not more than usual” (score 0), “little more than usual” (score 1), or “much more than usual” (score 1). Following questionnaire guidelines, a summed score ranged between 0 and 12, and a score ≥3 was categorized as “presence of psychological distress" [32].

### 2.3. Lifestyle and Weight Status

As dietary at risk variables, daily consumption (yes/no) of sugared soft drinks [34], of sweet or salty snacks (sum of candy, ice-cream, cookies, cake, chocolate, chips, etc.) and of less than five portions of fruit and vegetables (recommendation in several Western countries, in prevention of obesity [35,36]) were retrieved from a short food frequency questionnaire. Alcohol overconsumption during the last year was defined following the Belgian guideline, i.e., >14 drinks/week for women and >21 drinks/week for men (yes/no) [37]. Recent disordered eating was assessed via the five-item (yes/no) SCOFF (Sick, Control, One, Fat, Food) short screener [38] and scores ≥3 were categorized as at risk. Sleeping problems were retrieved via the Symptom CheckList-90-revised (SCL-90-R) [30] referring to the last week and using the mean of three items and categorizing those with total scores >1 as at risk. Lack of leisure time physical activity was categorized following the World Health Organization definition, i.e., <4 h/week light activity. Smoking was categorized as current smokers versus non-smokers. Excess weight was reflected as too high body mass index (BMI), i.e., overweight including obesity prevalence, based on self-reported weight and height, with BMI ≥ 25 for adults or using age- and gender-specific overweight cut-offs from Cole for youngsters [39].

### 2.4. Confounders

Age was recoded in four categories: 15–24 years (school-going youth and emerging adulthood), 25–44 years (young adulthood), 45–64 years (middle adulthood), ≥65 years (older adults, mostly retirees). Region of residence was coded as one of the 12 provinces. As a proxy for socio-economic status, the highest educational level within the household (Low “Higher secondary education or lower”, High “At least higher education”) and household income (relative to household composition) were considered. Two items covered physical health: “Do you suffer from one or more longstanding illnesses, chronic conditions or handicaps?” (yes/no) and “Do you have currently any problems in performing daily activities” (dichotomized as “no problems” versus "slight/moderate/severe/unable") from the EuroQol 5D-5L questionnaire [40].

### 2.5. Statistis

Statistics were performed using SPSS v23.0 (IBM, New York, NY, US) and mediation effects were obtained using the macro “Process”. Statistical significance was set at two-sided *p* < 0.05. Analyses accounted for the complex study design and were weighted to correct for differential response rates within the strata (the weight is the product of the reciprocal of the selection probability within a household and of a post stratification factor for each province according to age, gender, household size and quarter of the year in which the interview was done). Logistic regression analyses with lifestyle or excess weight as outcome were adjusted for age, gender, income, education, physical health parameters and stratification. All covariates were in categorical format as specified above and no interaction terms were included (since there was no significant model improvement based on chi-square of log-likelihoods). Odds ratios’ (OR) and their 95% confidence interval (CI) were shown as effect size.

Mediation is defined as a variable carrying the influence of a predictor to a given outcome and thus accounting for the observed relationship. Apart from significant predictor-mediator and mediator-outcome (adjusted for predictor) associations, the indirect effect should be significant (bootstrapping confidence intervals of the product-of-coefficients). To detect overall and independent lifestyle effects, a multiple mediation model was constructed with all lifestyle parameters added in the model together as mediator. As an effect size, the standardized indirect effect was used and the Pm was calculated as “indirect effect/total effect” reflecting how much of the total effect was explained by mediation.

Moderated mediation was tested, as age and gender differences appeared in the relation between mental health and lifestyle. Consequently, age and gender differences in the mediation between mental health and excess weight were tested.

## 3. Results

### 3.1. Descriptive Data

Characteristics of the study population can be found in Table 1. Excess weight was present in 46% of the population. From those who had high stress reports, 65.9% were also at risk for anxiety and/or depression. Supplemental Table 1 is showing age and sex differences. Women had a higher prevalence of stress, anxiety and/or depression, disordered eating, daily intake of at least five fruit and vegetable items and sleeping problems, but had a lower frequency of excess weight, daily soft drink intake, alcohol overconsumption, and lack of physical activity. Significant age differences were detected in all variables. The tested confounders were all significantly related to excess weight.

### 3.2. Relation of Mental Health with Lifestyle and Excess Weight

**Figure 1:**
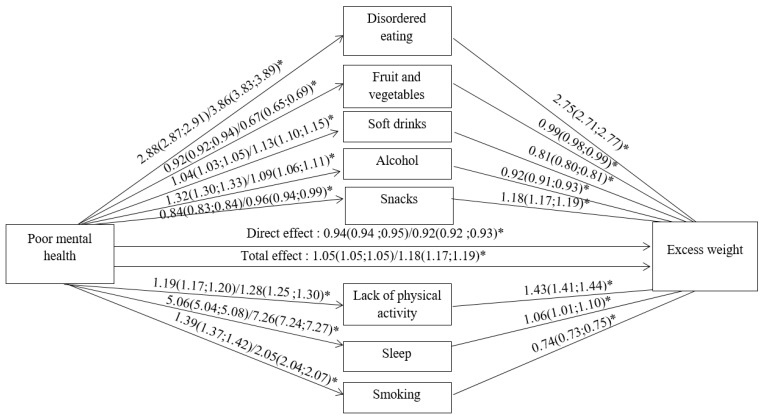
Logistic regression for associations between mental health, lifestyle, and excess weight. Odds ratios and 95% confidence intervals are shown as effect size: the first number is for stress, and the second is for anxiety and/or depression as mental health parameter. The total effect represents the association between mental health and excess weight without adjustment for lifestyle while the direct effect shows the association after adjustment for all lifestyle parameters. Analyses were adjusted for age, gender, income, education, and physical health, and weighted following the study design. * *p* < 0.05 (all were *p* < 0.01).

Without adjustment for physical health, stress (OR = 1.11; 95% CI (.10–1.11)) and anxiety and/or depression (OR = 1.26 (1.26–1.27)) were related to higher excess weight prevalence. After adjustment for physical health, the relations were still significant but weaker (OR = 1.05 (1.05–1.05) and OR = 1.18 (1.17–1.19), respectively). Figure 1 shows the relation (i.e., OR values) of mental health with lifestyle factors and excess weight for the total population, while Table 2 shows the OR stratified by gender and age. As a measure of mental health, the anxiety and/or depression measure showed larger effect sizes than the stress measure.

Table 2 indicates that several of these associations were gender- and age-specific. Only for disordered eating, smoking, and sleep, a consistent positive relation existed in all gender and age groups. Excess weight was most often positively related to stress or anxiety/depression, but a negative association was found in 45–64 years group and for stress in men and >65 years. Daily fruit and vegetables consumption had a consistent negative relation with anxiety and/or depression (OR = 0.67), while a positive relation with stress was seen in the younger groups (15–44 years) and men. Daily soft drink consumption was overall slightly (OR ≈ 1.1) higher in those with poor mental health (especially for anxiety and/or depression), but was lower in men (OR ≈ 0.8 to 0.9). Alcohol overconsumption was overall positively associated (OR ≈ 1.2) with poor mental health, but a negative association was seen in the youngest (15–24 years) and sometimes for anxiety and/or depression. In contrast to the hypothesis, the prevalence of daily snack intake was slightly lower in those with poor mental health (OR ≈ 0.9), while a positive relation was only found in the 15–24 years group (OR = 1.6). A lack of physical activity was positively related to poor mental health (OR ≈ 1.2) but this became non-significant for men and even negative for stress in ≥65 years and 25–44 years.

### 3.3. Mediation by Lifestyle in the Relation Between Mental Health and Excess Weight

Table 3 shows the indirect effects reflecting mediation capacity of the lifestyle factors. The overall model indicated a significant indirect effect: mediation by lifestyle explained at least 88% of the total effect. This large impact of the tested mediators is also reflected in Figure 1: while the total effect was significant and positive, the direct effect (after adding lifestyle factors in the regression between mental health and excess weight) was significantly negative. As can be seen in Table 3, disordered eating, physical activity, and smoking were significant independent mediators for both mental health measures. The strongest effect size was seen for disordered eating as positive mediator explaining at least 79% of the total effect. Lack of physical activity was also a positive mediator explaining at least 20% of the total effect. In contrast, smoking was a negative mediator explaining only 7% of the total effect: those with poor mental health were more frequent smokers, who consequently had less frequently excess weight.

### 3.4. Moderated Mediation: Depending on Age and Gender

Table 4 shows the significant moderated mediations: for age as moderator in the path via disordered eating, snacking, and smoking; and for gender as moderator in the path via smoking. Only the mediation by snacking changed significance depending on the moderator: snacking was a positive mediator in the youngest age group (β = 0.0501) but not in older age groups (β = 0.0004). The other mediations just changed in strength (but always stayed significant) depending on age or gender: the negative mediation by smoking was stronger in youth <30 years and women, and the positive mediation by disordered eating was stronger in youth.

## 4. Discussion

The data confirmed that poor mental health was significantly related to a higher excess weight prevalence (OR 1.11–1.26), even independent of physical health. The hypothesis of poor mental health being related to unhealthier lifestyle was also confirmed: more smoking, sleep problems, disordered eating, soft drink, and alcohol consumption, while less fruit and vegetables intake and less physical activity. Only the lower daily snack intake by mental health problems was in contrast to the hypothesis, but this was explained by the newly detected age differences. Indeed, gender- and age-specific differences were seen in the relation of mental health with excess weight and lifestyle: more health deterioration (such as snacking and excess weight) by poor mental health appeared for the youngest and women. Three lifestyle factors were significant mediators between poor mental health and excess weight: disordered eating and lack of physical activity as positive (=stimulating) mediators, while smoking as a previously unmentioned negative (= protective) mediator. The increased weight due to poor mental health disappeared and even became significantly negative after considering the lifestyle factors. Moderated mediation was present: negative mediation by smoking was stronger in women and the youngest; positive mediation by disordered eating was stronger in the youngest; and snacking was only a significant positive mediator in the youngest groups.

### 4.1. Diet and Disordered Eating

Stressed people may eat increased amounts of unhealthy food rich in sugar or fat because eating is a way to cope with stress [22] and stress can influence reward and appetite pathways [7,8,9,10] that increase the intake of unhealthier food and that decrease the intake of healthier food items. In the current study, disordered eating (mainly loss of control over eating) showed a very consistent positive pattern with high effect size, while the patterns for fruit and vegetables, snacks, and soft drinks were not perfectly consistent over all gender and age subgroups. Although an overall lower fruit and vegetables and higher soft drinks consumption confirmed the hypothesis, some opposite effects were found for men and the youngest groups. For daily snacking, the hypothesized positive relation was only found for youth (<25 years), while other groups showed a lowered snack intake by poor mental health.

The observed gender differences were in agreement with literature. Women tend to prefer the sweet or fatty food items as comfort food [41], while they also experience enhanced rewarding and appetitive emotional learning; this results in higher levels of craving and eating disorders [25]. Nevertheless, a study in 65,235 US adults did not find such gender difference [42]. The detected age difference in snacks corroborates a review where younger people scored snacks higher as comfort food [41]. Nevertheless, the observed lowered snack intake in older adults was not in agreement with literature in that age group [42,43]. Apart from stress-induced food intake, also stress-induced loss of appetite has been reported in the absence of palatable food, in people that are less sensitive to emotional eating or in the case of very intense or acute emotions [22,44]. Stress has been related to less eating occasions over the day in older adults [42]; this might explain the observed negative relation with snacks and fruit and vegetables in older groups. The positive association between mental health and fruit and vegetables in younger groups might be due to some people consuming fruit and vegetables as snack during stress-eating [41].

### 4.2. Physical Activity

Stressed people probably might not have the motivation nor the time to do physical activity, resulting in decreased activity by stress [11]. Indeed, mental health was related to a lack of physical activity (OR ≈ 1.2) in the current study, but this became non-significant for men and even a negative association for stress in ≥65 years. Depending on the age, people face different stressors. In emerging and young adults, work stress and childcare responsibilities might be highly time-consuming. In the retired population, time constraints are less present and physical activity might be used to cope with stress in a positive way, as a systematic review found several studies with stress-related elevations of physical activity [11]. Such positive stress coping has also been found in another extreme age group: the youngest children (with less time restraints) had a positive stress-activity association, while the older children decreased activity by stress [23]. Nevertheless, lower stress ratings were associated with increased physical activity in a natural experiment during retirement [45].

### 4.3. Sleep

Sleep duration and quality may be diminished by stress worrying, resulting in long sleep latency, many awakenings or short time in important sleep phases via hormonal and neuronal stress effects [13]. Research is very univocal about a positive relation between psychological and sleep disturbances [12]. In a population of low-income women, sleep seemed to be a stronger mediator than emotional eating in the depression–obesity relation [19]. Indeed, sleep problems displayed a very consistent positive pattern with mental health problems in the current study.

### 4.4. Smoking and Alcohol

Psychological stress can play a role in substance use initiation, maintenance, addiction, and relapse. After all, there are complex interactions between the biological stress response and the dopaminergic reward system but also by other systems crucial in moderating addiction-related behaviors such as endogenous opioids, the sympathetic-adrenal-medullary system, and endocannabinoids [46].

More frequent smoking by mental health problems was confirmed in the current study. Smokers may use nicotine to self-medicate depressed mood and fatigue since nicotine has been shown to stimulate positive mood by interactions with the dopaminergic and serotoninergic system [15]. In the current study, an even stronger relation was seen in women and youth <25 years. Indeed, higher vulnerability in women for stress-induced smoking has been reported [16]. Animal and clinical studies have suggested that women use nicotine to cope with anxiety to a larger extent than men, as women experience more biological stress-reducing and rewarding effects from smoking, probably due to estrogen differences [16].

Alcohol overconsumption was mostly increased (OR ≈ 1.2) by poor mental health, but decreased alcohol intake was seen in the youngest (15–24 years) and for the anxiety and or depression measure. Similar to smoking, drinking is often used to reduce stress or anxiety, possibly due to the sedative or depressant effect of alcohol on the nervous system. A systematic review identified seven studies with a positive significant association for men only, ten studies with a positive association for both genders, two studies with both positive and negative associations, and one study without significant association [14]. In a longitudinal study, increased health stressors predicted a reduction of alcohol consumption among women, while increased financial stressors suppressed alcohol consumption among men [47]. Similarly, a cross-sectional large study concluded that people under stressful conditions are more likely to either abstain or drink heavily rather than to drink lightly or moderately, depending on the stressor [48]. This explains the finding of both negative and positive associations. The lower vulnerability in youth for stress-induced drinking was not confirmed in an earlier stress coping study [24].

### 4.5. Excess Weight and Moderated Mediation

Overall, mental health problems were related to excess weight in the current study, even after adjusting for physical health. Generally, meta-analyses indeed confirmed a positive association for stress or depression with obesity [5,26]. In contrast, the current study detected a negative stress-weight relation in some subgroups: stress in the older groups (>44 years) and in men, and after adjusting for all lifestyle parameters. Indeed, a meta-analysis found a few studies with negative stress-weight associations [26]. Potential explanations for negative distress-weight associations are the observed gender- and age-differences and the consideration of lifestyle factors, e.g., smoking being a protective mediator (negatively associated with excess weight and positively with poor mental health).

Disordered eating, physical activity, and smoking were, in decreasing order, significant mediators. As most lifestyle relations with mental health were gender- or age-specific, moderated mediation was present. The mediation by smoking was strongest in women and youth <30 years, and daily snacking appeared as an additional positive mediator in youth <30 years since clear-cut emotion-induced snacking was visible only in youth. In literature, eating was the most frequent cited mediator [17,18,19,21,27], most studies only examined one or two lifestyle parameters and moderated mediation was seldom [18,27] tested. Consequently, comparisons with literature are difficult. In depression-related weight gain, stress eating, and sleep disturbance were significant independent mediators in women, while only sleep disturbance remained significant in a multiple mediation model [19]. Another study identified emotional eating and physical activity self-efficacy as mediators [21], and another found no mediation by external or restrained eating [18]. In post-traumatic stress disorder, binge-eating, but not alcohol use, was a mediator [20]. In university students, emotional eating and food addiction were significant mediators of stress [17]. Gender-specific mediation has also been reported in literature: emotional eating was only a mediator of depression-related overweight in women [18], and sedentary time was only a moderator of stressor effects in girls [27]. Further, in the current study, women were a high-risk group, but findings showed for the first time that especially age was important, with youth <25 years exhibiting more stress-induced snacking.

### 4.6. Strenghts and Limitations

Strengths are the availability of a representative sample of the Belgian population (using a weighing factor) with many lifestyle factors tested together to check which lifestyle factors are most influenced by mental health (both stress and anxiety and or depression). Novel is especially the moderated mediation analysis to check age- and gender-specific lifestyle changes in an attempt to explain conflicting results in different populations. Moreover, physical health was tested as confounder.

An important limitation is the use of self-reports: self-reported weight and height (potentially resulting in overweight underestimation), no stress biomarker and no objective lifestyle measures like accelerometers were implemented, although validated questionnaires were used. For soft drinks and snacks, only frequencies but no portion sizes were inquired. Most important, the cross-sectional data design (no temporal order) is not recommended in testing mediation since it does not allow to make a differentiation between testing confounding and mediation. Causality statements are even more problematic in the current topic since theoretical effects might be bidirectional both for excess weight and lifestyle, e.g., excess weight [5], diet [49] and physical activity [50] also influence mental health. Although multiple testing is an issue for Table 2, this is not an issue in multiple (moderated) mediation testing, as the model included all lifestyle factors in one analysis. Due to a large sample size, statistical significance was also reached for small effect sizes.

## 5. Conclusions and Translation towards Prevention and Intervention

The observed positive relation between mental health problems and excess weight indicates the importance of psychological interventions by stress management in obesity prevention programs. Although stress is not always inevitable, the way people cope with stress (i.e., emotion regulation skills) can be targeted as intervention [51]. As a reflection of stress coping, lifestyle explained 88% of the stress-weight associations and hence removed the positive relation between poor mental health and excess weight. Thus, lifestyle seems a good target in prevention and intervention. Herein, preventing loss of control over eating (as strongest mediator), decreasing high-calorie snacking, and stimulating physical activity seem the main targets based on the cross-sectional mediation. Prevention might then include, e.g., making people aware of these stress-induced changes, and stimulating the opposite direction by creating a healthy food environment and a physical activity motivating environment. The detected gender and age differences should be considered in clinical practice and group interventions. Especially youth seemed vulnerable for stress-related excess weight (while even negative associations were found for >45 years). The most clear-cut age difference was on snacking: the reduction of stress-induced snacking should thus especially be targeted in youth <25 years. Loss-of-control over eating had the highest mediation effect size towards excess weight in both men and women, although this might be reflected in other food items. Consequently, strengthening control over eating and mindful eating are suggested working points. On the contrary, stress might even be associated to a BMI decrease in older adults who exhibit less snacking. Although smoking partially (only 7%) attenuated the pathway to excess weight especially in women and youth <30 years, smoking should be discouraged in the perspective of other chronic diseases.

## Figures and Tables

**Table 1:** Descriptive data on the study population (*n* = 4687).

		*n*	%	% after Weighing
Age	15–24 years	495	10.6	12.1
	25–44 years	1637	34.9	34.9
	45–64 years	1652	35.2	34.8
	≥65 years	903	19.3	18.2
Gender	Men	2252	48.0	48.5
	Women	2435	52.0	51.5
Household equivalent income (quintiles in Belgium)	Lowest quintile	647	13.8	12.0
	Second quintile	689	14.7	14.6
	Third quintile	947	20.2	20.1
	Forth quintile	1083	23.1	24.8
	Highest quintile	1321	28.2	28.4
Highest educational level in household	No or primary	344	7.3	7.7
	Lower secondary	533	11.4	10.7
	Higher secondary	1428	30.5	32.6
	Higher	2382	50.8	49
Having a chronic condition		1360	29.0	26.4
Having a problem in performing daily activities		777	16.6	16.2
MENTAL HEALTH				
At risk for stress		1483	31.6	30.6
At risk for anxiety and/or depression		767	16.4	15.8
LIFESTYLE AND WEIGHT STATUS				
Excess weight (overweight or obesity)		2174	46.4	45.8
Disordered eating		376	8.0	7.5
Less than five portions of fruit and vegetables daily		671	14.3	13.0
Daily soft drink consumption		1031	22.0	23.1
Alcohol overconsumption		317	6.8	6.4
Daily snack intake		1841	39.3	40.1
Lack of physical activity		1313	28.0	73.2
Sleep problems		1340	28.6	28.7
Current smoking		1056	22.5	21.8

**Table 2:** Odds ratio for lifestyle factors and excess weight predicted by poor mental health (stress, anxiety, and/or depression), stratified by age and gender.

	Men		Women		15–24 Years		25–44 Years		45–64 Years		≥65 Years	
	Stress	Anxiety and/or Depression	Stress	Anxiety and/or Depression	Stress	Anxiety and/or Depression	Stress	Anxiety and/or Depression	Stress	Anxiety and/or Depression	Stress	Anxiety and/or Depression
Excess weight	0.95 (0.94–0.95)	1.18 (1.17–1.19)	1.11 (1.10–1.12)	1.19 (1.18–1.19)	1.02 (1.01–1.04)	2.16 (2.12–2.20)	1.12 (1.11–1.13)	1.22 (1.21–1.23)	0.9 (0.90–0.97)	0.99 (0.98–1.00)	0.78 (0.78–0.79)	1.06 (1.04–1.07)
Disordered eating	2.81 (2.77–2.83)	3.21 (3.16–3.24)	2.76 (1.73–2.78)	4.11 (4.07–4.14)	1.59 (1.56–1.62)	5.72 (5.60–5.83)	3.36 (3.33–3.40)	5.60 (5.53–5.66)	2.98 (2.94–3.02)	2.30 (1.11–1.13)	2.50 (2.45–2.55)	2.38 (2.33–2.43)
Daily fruit and vegetables	1.20 (1.19–1.22)	0.68 (0.66–0.69)	0.80 (0.79–0.80)	0.67 (0.66–0.68)	1.20 (1.17–1.23)	0.41 (0.39–0.43)	1.32 (1.31–1.33)	0.67 (0.66–0.68)	0.80 (0.79–0.80)	0.82 (0.80–0.83)	0.53 (0.52–0.53)	0.33 (0.31–0.33)
Daily soft drinks	0.93 (0.93–0.94)	0.87 (0.85–0.87)	1.19 (1.17–1.19)	1.36 (1.34–1.36)	0.89 (0.88–0.91)	1.26 (1.24–1.28)	0.83 (0.82–0.83)	1.03 (1.01–1.03)	1.14 (1.13–1.14)	1.16 (1.15–1.17)	1.74 (1.71–1.76)	1.17 (1.15–1.19)
Alcohol overconsumption	1.23 (1.21–1.24)	1.18 (1.16–1.19)	1.41 (1.39–1.42)	0.67 (0.65–0.68)	0.41 (0.39–0.42)	0.43 (0.41–0.45)	1.38 (1.35–1.40)	0.92 (0.89–0.94)	1.51 (1.49–1.52)	1.04 (1.02–1.05)	1.31 (1.28–1.34)	0.68 (0.66–0.70)
Daily snacks	0.75 (0.75–0.75)	0.91 (0.89–0.91)	0.95 (0.95–0.96)	0.98 (0.97–0.98)	1.05 (1.04–1.08)	1.59 (1.56–1.61)	0.77 (0.76–0.77)	0.92 (0.91–0.92)	0.75 (0.75–0.76)	0.87 (0.86–0.88)	1.16 (1.15–1.17)	0.88 (0.87–0.89)
Lack of physical activity	0.99 ^NS^ (0.99–1.01)	1.00 ^NS^ (0.99–1.01)	1.17 (1.16–1.18)	1.49 (1.48–1.50)	1.62 (1.60–1.65)	1.71 (1.68–1.74)	0.98 (0.97–0.99)	1.37 (1.36–1.38)	1.30 (1.28–1.31)	1.23 (1.21–2.24)	0.84 (0.83–0.85)	1.35 (1.33–1.36)
Sleep problems	6.21 (6.17–6.25)	8.49 (8.41–8.57)	4.36 (4.33–4.38)	6.77 (6.72–6.82)	4.41 (4.33–4.48)	6.10 (6.01–6.18)	4.46 (4.42–4.49)	7.31 (7.23–7.37)	6.69 (6.64–6.73)	12.066 (11.94–12.19)	3.75 (3.71–3.78)	4.56 (4.50–4.61)
Current smoking	1.21 (1.20–1.22)	1.40 (1.38–1.41)	1.57 (1.16–1.58)	2.61 (2.59–2.62)	2.10 (2.07–2.12)	3.78 (3.71–3.84)	1.12 (1.11–1.32)	1.63 (1.61–1.64)	1.20 (1.19–1.31)	1.81 (1.79–1.82)	1.60 (1.57–1.62)	1.85 (1.81–1.88)

Logistic regression analyses were adjusted for age or gender and income, education, and physical health; and weighted following the study design. NS = non-significant, *p* > 0.05.

**Table 3:** Standardized indirect effects of lifestyle mediation in the relation between mental health (stress or anxiety and/or depression) and excess weight.

	Stress	Anxiety and/or Depression
All mediators	0.056 * (0.009; 0.075) Pm = 0.88	0.061 * (0.039; 0.083) Pm = 0.93
Disordered eating	0.050 * (0.040; 0.061) Pm = 0.79	0.061 * (0.049; 0.074) Pm = 0.83
Daily fruit and vegetables	−0.001 (−0.003; 0.002) Pm < −0.01	−0.001 (−0.004; 0.003) Pm < −0.01
Daily soft drinks	0.000 (−0.001; 0.001) Pm < 0.01	−0.001 (−0.001; 0.001) Pm < −0.01
Alcohol overconsumption	−0.001 (−0.002; 0.001) Pm< −0.01	−0.001 (−0.001; 0.001) Pm < −0.01
Daily snacks	0.001 (−0.001; 0.004) Pm = 0.02	0.001 (−0.001; 0.003) Pm = 0.01
Lack of physical activity	0.010 * (0.006; 0.015) Pm = 0.20	−0.014 * (0.009; 0.019) Pm = 0.21
Sleep problems	−0.001 (−0.014; 0.014) Pm = −0.01	−0.008 (−0.022; 0.009) Pm =-0.11
Current smoking	−0.005 * (−0.008; −0.002) Pm = −0.07	−0.007* (−0.012; −0.004) Pm = −0.10

Standardized indirect effect regression coefficient and bootstrapped 95% confidence interval are shown together with Pm (“indirect effect/total effect”). Analyses were adjusted for age, gender, income, education, physical health, and weighted following the study design. * 0 is not in the 95% confidence interval; thus, the indirect effect was significant.

**Table 4:** Standardized indirect effect for lifestyle mediation with significant moderated mediation by age or gender in the relation between mental health (anxiety and/or depression) and excess weight.

Mediator: Disordered Eating; Moderator: Age		
Indirect Effect		Direct Effect
−1SD age (30 years)	0.06(0.04; 0.07) *	0.98 (0.97; 0.99) *
mean age (49 years)	0.04(0.03; 0.06) *	1.00 (0.99; 1.02)
+1SD age (68 years)	0.03(0.01; 0.04) *	0.99 (0.98; 1.01)
Mediator: daily snacks; Moderator: age		
−1SD age (30 years)	0.05(0.02; 0.07) *	1.26 (1.25; 1.27) *
mean age (49 years)	0.01(0.00; 0.01) *	1.11 (1.10; 1.12) *
+1SD age (68 years)	0.01(−0.01; 0.03)	1.12 (1.11; 1.14) *
Mediator: current smoking; Moderator: age		
−1SD age (30 years)	−0.02(−0.05; −0.01) *	1.30 (1.28; 1.31) *
mean age (49 years)	−0.01(−0.03; −0.01) *	1.17 (1.16; 1.18) *
+1SD age (68 years)	−0.01(−0.02; −0.01 *	1.12 (1.11; 1.13) *
Mediator: current smoking; Moderator: gender		
Men	−0.01(−0.01; −0.01) *	1.31 (1.30; 1.32) *
Women	−0.02(−0.02; −0.01) *	1.26 (1.25; 1.27) *

Standardized indirect effect with bootstrapped 95% confidence intervals and direct effect are shown. Analyses were adjusted for age or gender (depending on tested moderator) and income, education, physical health, and weighted following the study design. * 0 is not in the 95% confidence interval of the indirect effect, thus reflecting a significant effect. SD = standard deviation.

## Data Availability

The authors do not own the data but paid an access fee, data belongs to the Scientific Institute of Public health, see https://his.wiv-isp.be/nl/SitePages/Procedure_gegevens2013.aspx.
